# Supplementary material for: HIVprotI: an integrated web based platform for prediction and design of HIV proteins inhibitors
Source: J Cheminform. 2018 Mar 9;10:12. doi: 10.1186/s13321-018-0266-y (PMC5845081; doi:10.1186/s13321-018-0266-y)
Supplement: Supplementary file 2 — Additional file 2. Source code of HIVProtI web server. [file 13321_2018_266_MOESM2_ESM.zip › HIVprotI_Source-code/hivprotI/weka-3-6-6/doc/weka/classifiers/CostMatrix.html]

CostMatrix


---


|  |  |  |
| --- | --- | --- |
| **Overview** \| **Package** \| **Class** \| **Tree** \| **Deprecated** \| **Index** \| **Help** \| **Weka's home** | |  |
| **PREV CLASS**   **NEXT CLASS** | **FRAMES**    **NO FRAMES**     **All Classes** |  |
| SUMMARY: NESTED \| FIELD \| CONSTR \| METHOD | DETAIL: FIELD \| CONSTR \| METHOD |  |


---


## weka.classifiers Class CostMatrix

```
java.lang.Object
  weka.classifiers.CostMatrix
```

**All Implemented Interfaces:**: java.io.Serializable, RevisionHandler

---

``` public class CostMatrix extends java.lang.Object implements java.io.Serializable, RevisionHandler ```

Class for storing and manipulating a misclassification cost matrix.
The element at position i,j in the matrix is the penalty for classifying
an instance of class j as class i. Cost values can be fixed or
computed on a per-instance basis (cost sensitive evaluation only)
from the value of an attribute or an expression involving
attribute(s).

**Version:**
:   $Revision: 1.17 $

**Author:**
:   Mark Hall, Richard Kirkby (rkirkby@cs.waikato.ac.nz)

**See Also:**: Serialized Form

---

| **Field Summary** | |
| --- | --- |
| `static java.lang.String` | `FILE_EXTENSION`             The deafult file extension for cost matrix files |


**Constructor Summary**

`CostMatrix(CostMatrix toCopy)`
  
          Creates a cost matrix that is a copy of another.

`CostMatrix(int numOfClasses)`
  
          Creates a default cost matrix of a particular size.

`CostMatrix(java.io.Reader reader)`
  
          Reads a matrix from a reader.


| **Method Summary** | |
| --- | --- |
| `Instances` | `applyCostMatrix(Instances data, java.util.Random random)`             Applies the cost matrix to a set of instances. |
| `double[]` | `expectedCosts(double[] classProbs)`             Calculates the expected misclassification cost for each possible class value, given class probability estimates. |
| `double[]` | `expectedCosts(double[] classProbs, Instance inst)`             Calculates the expected misclassification cost for each possible class value, given class probability estimates. |
| `java.lang.Object` | `getCell(int rowIndex, int columnIndex)`             Return the contents of a particular cell. |
| `double` | `getElement(int rowIndex, int columnIndex)`             Return the value of a cell as a double (for legacy code) |
| `double` | `getElement(int rowIndex, int columnIndex, Instance inst)`             Return the value of a cell as a double. |
| `double` | `getMaxCost(int classVal)`             Gets the maximum cost for a particular class value. |
| `double` | `getMaxCost(int classVal, Instance inst)`             Gets the maximum cost for a particular class value. |
| `java.lang.String` | `getRevision()`             Returns the revision string. |
| `void` | `initialize()`             Initializes the matrix |
| `void` | `normalize()`             Normalizes the matrix so that the diagonal contains zeros. |
| `int` | `numColumns()`             Same as size |
| `int` | `numRows()`             Same as size |
| `static Matrix` | `parseMatlab(java.lang.String matlab)`             creates a matrix from the given Matlab string. |
| `void` | `readOldFormat(java.io.Reader reader)`             Loads a cost matrix in the old format from a reader. |
| `void` | `setCell(int rowIndex, int columnIndex, java.lang.Object value)`             Set the value of a particular cell in the matrix |
| `void` | `setElement(int rowIndex, int columnIndex, double value)`             Set the value of a cell as a double |
| `int` | `size()`             The number of rows (and columns) |
| `java.lang.String` | `toMatlab()`             converts the Matrix into a single line Matlab string: matrix is enclosed by parentheses, rows are separated by semicolon and single cells by blanks, e.g., [1 2; 3 4]. |
| `java.lang.String` | `toString()`             Converts a matrix to a string. |
| `void` | `write(java.io.Writer w)`             Writes out a matrix. |

**Methods inherited from class java.lang.Object**

`equals, getClass, hashCode, notify, notifyAll, wait, wait, wait`

**Field Detail**

### FILE\_EXTENSION

```
public static java.lang.String FILE_EXTENSION
```

:   The deafult file extension for cost matrix files


**Constructor Detail**

### CostMatrix

```
public CostMatrix(int numOfClasses)
```

:   Creates a default cost matrix of a particular size.
    All diagonal values will be 0 and all non-diagonal values 1.

    **Parameters:**: `numOfClasses` - the number of classes that the cost matrix holds.

---


### CostMatrix

```
public CostMatrix(CostMatrix toCopy)
```

:   Creates a cost matrix that is a copy of another.

    **Parameters:**: `toCopy` - the matrix to copy.

---


### CostMatrix

```
public CostMatrix(java.io.Reader reader)
           throws java.lang.Exception
```

:   Reads a matrix from a reader. The first line in the file should
    contain the number of rows and columns. Subsequent lines
    contain elements of the matrix.
    (FracPete: taken from old weka.core.Matrix class)

    **Parameters:**: `reader` - the reader containing the matrix **Throws:**: `java.lang.Exception` - if an error occurs **See Also:**: `write(Writer)`


**Method Detail**

### initialize

```
public void initialize()
```

:   Initializes the matrix

---


### size

```
public int size()
```

:   The number of rows (and columns)

    :   **Returns:**: the size of the matrix

---


### numColumns

```
public int numColumns()
```

:   Same as size

    :   **Returns:**: the number of columns

---


### numRows

```
public int numRows()
```

:   Same as size

    :   **Returns:**: the number of rows

---


### applyCostMatrix

```
public Instances applyCostMatrix(Instances data,
                                 java.util.Random random)
                          throws java.lang.Exception
```

:   Applies the cost matrix to a set of instances. If a random number generator is
    supplied the instances will be resampled, otherwise they will be rewighted.
    Adapted from code once sitting in Instances.java

    :   **Parameters:**: `data` - the instances to reweight.: `random` - a random number generator for resampling, if null then instances are rewighted. **Returns:**: a new dataset reflecting the cost of misclassification. **Throws:**: `java.lang.Exception` - if the data has no class or the matrix in inappropriate.

---


### expectedCosts

```
public double[] expectedCosts(double[] classProbs)
                       throws java.lang.Exception
```

:   Calculates the expected misclassification cost for each possible class value,
    given class probability estimates.

    :   **Parameters:**: `classProbs` - the class probability estimates. **Returns:**: the expected costs. **Throws:**: `java.lang.Exception` - if the wrong number of class probabilities is supplied.

---


### expectedCosts

```
public double[] expectedCosts(double[] classProbs,
                              Instance inst)
                       throws java.lang.Exception
```

:   Calculates the expected misclassification cost for each possible class value,
    given class probability estimates.

    :   **Parameters:**: `classProbs` - the class probability estimates.: `inst` - the current instance for which the class probabilites apply. Is used for computing any non-fixed cost values. **Returns:**: the expected costs. **Throws:**: `java.lang.Exception` - if something goes wrong

---


### getMaxCost

```
public double getMaxCost(int classVal)
                  throws java.lang.Exception
```

:   Gets the maximum cost for a particular class value.

    :   **Parameters:**: `classVal` - the class value. **Returns:**: the maximum cost. **Throws:**: `java.lang.Exception` - if cost matrix contains non-fixed costs

---


### getMaxCost

```
public double getMaxCost(int classVal,
                         Instance inst)
                  throws java.lang.Exception
```

:   Gets the maximum cost for a particular class value.

    :   **Parameters:**: `classVal` - the class value. **Returns:**: the maximum cost. **Throws:**: `java.lang.Exception` - if cost matrix contains non-fixed costs

---


### normalize

```
public void normalize()
```

:   Normalizes the matrix so that the diagonal contains zeros.

---


### readOldFormat

```
public void readOldFormat(java.io.Reader reader)
                   throws java.lang.Exception
```

:   Loads a cost matrix in the old format from a reader. Adapted from code once sitting
    in Instances.java

    :   **Parameters:**: `reader` - the reader to get the values from. **Throws:**: `java.lang.Exception` - if the matrix cannot be read correctly.

---


### write

```
public void write(java.io.Writer w)
           throws java.lang.Exception
```

:   Writes out a matrix. The format can be read via the
    CostMatrix(Reader) constructor.
    (FracPete: taken from old weka.core.Matrix class)

    :   **Parameters:**: `w` - the output Writer **Throws:**: `java.lang.Exception` - if an error occurs

---


### toMatlab

```
public java.lang.String toMatlab()
```

:   converts the Matrix into a single line Matlab string: matrix is enclosed
    by parentheses, rows are separated by semicolon and single cells by
    blanks, e.g., [1 2; 3 4].

    :   **Returns:**: the matrix in Matlab single line format

---


### setCell

```
public final void setCell(int rowIndex,
                          int columnIndex,
                          java.lang.Object value)
```

:   Set the value of a particular cell in the matrix

    :   **Parameters:**: `rowIndex` - the row: `columnIndex` - the column: `value` - the value to set

---


### getCell

```
public final java.lang.Object getCell(int rowIndex,
                                      int columnIndex)
```

:   Return the contents of a particular cell. Note: this
    method returns the Object stored at a particular cell.

    :   **Parameters:**: `rowIndex` - the row: `columnIndex` - the column **Returns:**: the value at the cell

---


### getElement

```
public final double getElement(int rowIndex,
                               int columnIndex)
                        throws java.lang.Exception
```

:   Return the value of a cell as a double (for legacy code)

    :   **Parameters:**: `rowIndex` - the row: `columnIndex` - the column **Returns:**: the value at a particular cell as a double **Throws:**: `java.lang.Exception` - if the value is not a double

---


### getElement

```
public final double getElement(int rowIndex,
                               int columnIndex,
                               Instance inst)
                        throws java.lang.Exception
```

:   Return the value of a cell as a double. Computes the
    value for non-fixed costs using the supplied Instance

    :   **Parameters:**: `rowIndex` - the row: `columnIndex` - the column **Returns:**: the value from a particular cell **Throws:**: `java.lang.Exception` - if something goes wrong

---


### setElement

```
public final void setElement(int rowIndex,
                             int columnIndex,
                             double value)
```

:   Set the value of a cell as a double

    :   **Parameters:**: `rowIndex` - the row: `columnIndex` - the column: `value` - the value (double) to set

---


### parseMatlab

```
public static Matrix parseMatlab(java.lang.String matlab)
                          throws java.lang.Exception
```

:   creates a matrix from the given Matlab string.

    :   **Parameters:**: `matlab` - the matrix in matlab format **Returns:**: the matrix represented by the given string **Throws:**: `java.lang.Exception`

---


### toString

```
public java.lang.String toString()
```

:   Converts a matrix to a string.
    (FracPete: taken from old weka.core.Matrix class)

    :   **Overrides:**: `toString` in class `java.lang.Object`
    :   **Returns:**: the converted string

---


### getRevision

```
public java.lang.String getRevision()
```

:   Returns the revision string.

    :   **Specified by:**: `getRevision` in interface `RevisionHandler`
    :   **Returns:**: the revision


---


|  |  |  |
| --- | --- | --- |
| **Overview** \| **Package** \| **Class** \| **Tree** \| **Deprecated** \| **Index** \| **Help** \| **Weka's home** | |  |
| **PREV CLASS**   **NEXT CLASS** | **FRAMES**    **NO FRAMES**     **All Classes** |  |
| SUMMARY: NESTED \| FIELD \| CONSTR \| METHOD | DETAIL: FIELD \| CONSTR \| METHOD |  |


---
